# Associations of estimated glucose disposal rate with kidney stones in U.S. non-diabetic adults and possible mediating mechanisms: NHANES 2009–2020

**DOI:** 10.1371/journal.pone.0328576

**Published:** 2025-07-29

**Authors:** Haowen Liang, Ying Wei

**Affiliations:** 1 Department of Urology, Liuzhou Traditional Chinese Medical Hospital, Liuzhou, Guangxi, China; 2 Department of Nephrology, Liuzhou Traditional Chinese Medical Hospital, Liuzhou, Guangxi, China; Ferdowsi University of Mashhad, IRAN, ISLAMIC REPUBLIC OF

## Abstract

**Background:**

Kidney stone formation has been linked to insulin resistance (IR). However, the association between the estimated glucose disposal rate (eGDR) – a novel surrogate marker for IR – and kidney stone occurrence in non-diabetic adults remains unclear.

**Methods:**

We analyzed data from adult participants in the National Health and Nutrition Examination Survey (NHANES) collected between 2009 and 2020 who self-reported a history of kidney stones. To assess the relationship between eGDR and kidney stones, we applied a range of statistical methods, including weighted proportions, multivariable logistic regression, restricted cubic splines (RCS), receiver operating characteristic (ROC) curve analysis, subgroup analysis, and mediation analysis.

**Results:**

The final analysis included 8,051 participants, of whom 8.71% reported a history of kidney stones. Multivariable logistic regression revealed that, compared to the lowest eGDR quartile, the fully adjusted odds ratios (95% confidence intervals) for kidney stone in the second, third, and fourth quartiles were 0.87 (0.61–1.26), 0.54 (0.34–0.85), and 0.46 (0.28–0.77), respectively. The RCS plot indicated a significant non-linear inverse association between eGDR and kidney stone risk. ROC curve analysis showed that the association between eGDR and the risk of kidney stones was more pronounced compared to the other five IR indicators, as evidenced by a higher area under the curve. Mediation analysis identified albumin (ALB) and red cell distribution width (RDW) as partial mediators in the association between IR and kidney stones.

**Conclusion:**

Our research results indicate that lower levels of eGDR are associated with an increased risk of developing kidney stones in non-diabetic adults. Furthermore, ALB and RDW may partially mediate the relationship between IR and kidney stones.

## Introduction

Kidney stones represent a common urological disorder with a high prevalence, frequent recurrence, significant hospitalization rates, and substantial treatment costs. Together, these factors impose a considerable burden on patients, their families, and the healthcare system [1,2]. Typically, kidney stones form in the kidneys due to either increased urinary concentrations or reduced solubility of crystallizable substances. These conditions lead to urinary supersaturation, promoting crystal precipitation, aggregation, and subsequent stone formation. Although most individuals with kidney stones remain asymptomatic, a subset may present with renal colic resulting from ureteral obstruction by stones [3]. Without timely medical intervention, kidney stones can lead to serious complications, including urogenital sepsis, septic shock, renal abscesses, and chronic kidney disease, and in severe cases, may become life-threatening [4–6]. Given these risks, identifying modifiable and non-modifiable risk factors for kidney stones is essential for early intervention, effective management, and prevention of adverse outcomes.

Kidney stones are not a single-factor disease; rather, it involves multiple contributing factors and reflects a systemic metabolic disorder [7]. Although the precise mechanisms underlying kidney stones remain incompletely understood, accumulating evidence suggests that insulin resistance (IR) may act as a significant risk factor for its development [8,9]. IR is a hallmark of several metabolic conditions, including obesity, prediabetes, and type 2 diabetes. It impairs renal tubular ammonia production, disrupts metabolic homeostasis, and compromises overall kidney function. Moreover, IR is closely associated with elevated systemic inflammation and oxidative stress, both of which contribute to renal pathology [10]. Kidney stones frequently coexist with metabolic syndrome, dyslipidemia, and abdominal obesity—conditions strongly linked to IR [11,12]. Current evidence indicates that IR promotes kidney stone formation by impairing renal tubular function and altering urinary composition and pH levels [13]. Multiple indices have been developed to quantify IR, many of which are associated with kidney stone risk. These include the metabolic score for insulin resistance (METS-IR), triglyceride-glucose body mass index (TyG-BMI), triglyceride-glucose waist-to-height ratio (TyG-WHtR), triglyceride-glucose waist circumference (TyG-WC), and the triglyceride-to-high-density lipoprotein cholesterol ratio (TG/HDL-C). These markers consistently show that individuals—particularly males and those with diabetes—face a heightened risk of developing kidney stones [14,15]. However, several recently proposed markers of insulin resistance, including the estimated glucose disposal rate (eGDR) and the ratio of glycosylated hemoglobin A1c to high-density lipoprotein cholesterol (HbA1c/HDL-C), have not yet been evaluated in relation to kidney stone development. Therefore, this cross-sectional study aims to identify the most effective indicator of IR for assessing its association with kidney stones in a non-diabetic population.

Albumin (ALB), synthesized primarily in the liver, constitutes approximately 55% of total plasma protein. In states of IR, hepatic protein synthesis is impaired, resulting in reduced albumin production. ALB plays several critical physiological roles, including maintaining plasma colloid osmotic pressure, buffering acids and bases, preserving antioxidant capacity, and modulating extracellular sulfhydryl groups [16]. A decline in ALB levels may compromise the body’s ability to counteract oxidative stress. Additionally, red cell distribution width (RDW) has been strongly linked to intracellular oxidative stress. Elevated RDW levels typically reflect increased oxidative stress burden [17]. Given the established connection between oxidative stress and kidney stone formation [18], we hypothesize that ALB and RDW may serve as key mediators in the relationship between IR and kidney stones.

Accordingly, this study investigates the association between eGDR and kidney stones among non-diabetic U.S. adults and explores the potential mediating roles of ALB and RDW in this relationship. These mediators may offer insight into the role of oxidative stress in linking insulin resistance to kidney stone formation.

## Methods

### Study population

**Fig 1:**
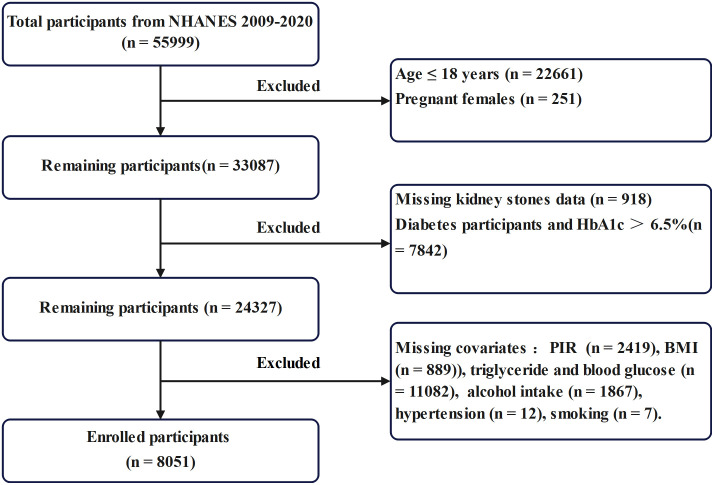
Participants and flowcharts.

We obtained data for this study from NHANES, which uses a stratified, multistage probability sampling design to ensure nationally representative estimates. We included data from six NHANES cycles spanning 2009–2020. The National Center for Health Statistics (NCHS) Research Ethics Review Board approved all NHANES protocols involving human participants, and all individuals provided written informed consent. We included participants with complete data on fasting blood glucose (FBG), triglycerides (TG), high-density lipoprotein cholesterol (HDL-C), glycosylated hemoglobin A1c (HbA1c), waist circumference (WC), height, ALB, and RDW, along with complete diagnostic information on kidney stone history. We excluded individuals aged ≤18 years, pregnant participants, those with diabetes or HbA1c > 6.5%, and anyone with missing data for the required variables. After applying these criteria, we included a total of 8,051 participants in the final analysis. The participant selection process is detailed in Fig 1. All participants provided informed consent. As our study used publicly available, de-identified data and involved no direct patient intervention, ethical approval was not required by the Liuzhou Traditional Chinese Medicine Hospital, in accordance with the principles of the Declaration of Helsinki.

### Six different insulin resistance indicators

We calculated six alternative indices of IR using the following formulas:

TyG-WHtR [19]:


$$\begin{array}{c} TyG=Ln\;[fasting\;triglyceride\;(mg/dL)\times fasting\;glucose\;(mg/dL)/2] \end{array}$$

TyG−WHtR=TyG×[WC(cm)/WC(cm)Height\nulldelimiterspaceHeight(cm)]

TyG-BMI [14]:


$$\begin{array}{c} TyG-BMI=BMI\times TyG\;index,where\;BMI=weight/height^{2} \end{array}$$

TyG-WC [20]:


$$\begin{array}{c} TyG-WC=TyG\;index\times WC(cm) \end{array}$$

METS-IR [14]:


$$\begin{array}{c} METS-IR=\frac{Ln[(2\times fasting\;triglyceride\;(mg/dL)+fasting\;triglyceride\;(mg/dL))\times BMI]}{Ln[HDL-C(mg/dL)]} \end{array}$$

HbA1c/HDL-C [21]:


HbA1c/HDL−C=HbA1c(%)/HDL−C(mmol/L)

eGDR [22]:


eGDR=21.158−(0.09×WC(cm))−(3.407×Hypertension)−(0.551×HbA1c)

(Hypertension: yes = 1, no = 0; HbA1c (%).

### Screening of kidney stones

We assessed participants’ history of kidney stones using a structured questionnaire focused on renal health. Specially trained interviewers conducted one-on-one interviews to collect this information. We classified participants as having a history of kidney stones if they responded “yes” to the relevant question.

### Determination of covariables

We collected demographic data, including gender, age, race/ethnicity, marital status, educational attainment, and the poverty-income ratio, through interview questionnaires. Smoking status was categorized as follows: current (smoking at least one cigarette per day), never (smoking fewer than 100 cigarettes in a lifetime), and former (smoking more than 100 cigarettes but no longer smoking). Alcohol consumption was classified as: yes (consumption of more than 12 alcoholic beverages per year) and no (12 or fewer alcoholic beverages per year). Body mass index (BMI) and WC were measured using a body measurement board. Hypertension status was determined via a questionnaire, with responses restricted to “yes” or “no,” excluding missing or invalid answers. Vigorous recreational activity, defined as activities that significantly elevate breathing or heart rate for at least 10 minutes weekly, included running and playing basketball. Dietary information was gathered through two 24-hour dietary recall interviews, focusing on sodium, calcium, and water intake. We calculated the average daily intake of these dietary components. We obtained laboratory data, including FBG, TG, HDL-C, glycosylated HbA1c, creatinine (Cr), uric acid (UA), ALB, and RDW from laboratory records. Detailed descriptions of the laboratory tests are publicly available on the NHANES website (www.cdc.gov/nchs/nhanes/). We estimated glomerular filtration rate (eGFR) using the Chronic Kidney Disease Epidemiology Collaboration (CKD-EPI) formula [23]:


 eGFR = 142 × (Scr/A) ^  B ×(0.9938) ^  age × C(Scr:serum creatinine)

Female: C = 1.012; Scr ≤ 0.7 mg/dL, A = 0.7, B = −0.241; Scr > 0.7 mg/dL, A = 0.7, B = −1.2;

Male: C = 1.012; Scr ≤ 0.9 mg/dL, A = 0.9, B = −0.302; Scr > 0.9 mg/dL, A = 0.7, B = −1.2.

### Statistical analysis

Statistical analyses were conducted using sample weights from NHANES, in accordance with NHANES guidelines, which account for the complex, multistage design of the cohort. Due to the COVID-19 pandemic, which lasted for 39 months, the 2017–2020 cycle was prematurely concluded in March 2020. Given that one cycle spans 24 months, the 2009–2016 period corresponds to four cycles (96 months/ 24 months), while the 2017 to early 2020 period represents approximately 1.625 cycles (39 months/ 24 months). Consequently, the weights for participants from 2009 to 2016 were calculated as 1/ (1.625 + 4) × Fasting Subsample 2 Year MEC Weight (WTSAF2YR), while those for participants from 2017 to early 2020 were calculated as 1.625/ (1.625 + 4) × Fasting Subsample Weight (WTSAFPRP). Categorical variables were expressed as percentages, and continuous variables were presented as means with standard deviations (SD). Differences between groups were assessed using one-way ANOVA, unpaired t-tests, and chi-squared tests, as appropriate. Logistic regression was employed to estimate the odds ratios (OR) and 95% confidence intervals (CI) for the association between the eGDR and kidney stones. The eGDR was divided into quartiles (Q1, Q2, Q3, Q4). We used three statistical models to examine the relationship between the eGDR and kidney stones.

Model 1 excluded all covariates. Model 2 adjusted for factors such as gender, age, race, education, poverty ratio, and marital status. Model 3 further included hypertension, alcohol consumption, smoking, vigorous activity, creatinine, and uric acid. We explored the dose-response and linear relationships between the eGDR and kidney stones using restricted cubic splines. Additionally, we assessed the correlation between the six IR indicators and kidney stones by calculating the area under the ROC curve. To explore potential heterogeneity in the association between the eGDR and kidney stones, we conducted subgroup analyses considering variables such as gender, age, race, BMI, education, poverty ratio, marital status, hypertension, alcohol intake, smoking, and vigorous activity. We also performed mediation analyses, adjusting for all confounding variables, to quantify the mediation effect. The proportion of mediation represents the percentage of the total effect mediated by specific variables. The significance of the mediation effect was assessed using bootstrap sampling (iterations = 1000) [24].

We used DecisionLinnc1.0 software for data analysis [25]. This platform integrates multiple programming languages and enables data analysis and machine learning through a user-friendly graphical interface. A *p*-value* *< 0.05 was considered statistically significant.

## Results

### Baseline characteristics of participants

Table 1 presents the demographic and social characteristics, as well as the laboratory results, of the study participants. Our analysis included 8,051 individuals, comprising 701 with kidney stones and 7,350 without kidney stones. We found significant differences between the two groups in terms of age, WC, eGFR, RDW, BMI, FBG, TG, HDL-C, HbA1c, race, hypertension, vigorous physical activity, and marital status (*p* < 0.05). However, no significant differences were observed between the groups with regard to gender, education, smoking status, alcohol consumption frequency, poverty-to-income ratio (PIR), creatinine levels, or uric acid levels. The data showed that individuals with kidney stones had higher levels of TyG-WHtR, TyG-BMI, TyG-WC, METS IR, and the HbA1c/HDL-C ratio, while their eGDR levels were lower (*p* < 0.05).

**Table 1:** Demographic characteristics of the study participants, weighted.

Characteristic	Total (n = 8051)	kidney stones status		*p*-value
		Controls(n = 7350)	Cases(n = 701)	
**General characteristics**				
Gender, n (%)				0.235
Female	3853 (48.98%)	3535 (49.24%)	318 (46.48%)	
Male	4198 (51.02%)	3815 (50.76%)	383 (53.52%)	
Age (years)	46.14 ± 16.50	45.64 ± 16.51	51.01 ± 15.61	< 0.001
Height (cm)	169.60 ± 9.75	169.65 ± 9.69	169.08 ± 10.37	0.160
Waist circumference (cm)	98.56 ± 15.05	98.16 ± 15.01	102.46 ± 14.92	< 0.001
BMI (kg/m^2^)	28.72 ± 6.09	28.60 ± 6.08	29.88 ± 6.09	< 0.001
Dietary calcium (mg/day)	982.50 ± 483.23	984.65 ± 483.31	961.39 ± 482.30	0.452
Dietary sodium (mg/day)	3532.50 ± 1398.41	3533.38 ± 1391.45	3523.89 ± 1465.94	0.606
Dietary water (gm/day)	3009.41 ± 1234.80	3016.33 ± 1245.37	2941.65 ± 1124.63	0.382
Race, n (%)				< 0.001
Mexican American	1045 (7.65%)	963 (7.82%)	82 (5.93%)	
Other Hispanic	797 (5.50%)	725 (5.54%)	72 (5.19%)	
Non-Hispanic White	3698 (70.40%)	3291 (69.63%)	407 (77.90%)	
Non-Hispanic Black	1560 (9.67%)	1481 (10.18%)	79 (4.65%)	
Other race	951 (6.78%)	890 (6.83%)	61 (6.33%)	
Educational attainment, n (%)				0.853
College grade	4788 (66.42%)	4364 (66.47%)	424 (65.92%)	
High school grade	1788 (21.62%)	1643 (21.66%)	145 (21.30%)	
Lower than 12th grade	1475 (11.96%)	1343 (11.87%)	132 (12.78%)	
Marital status, n (%)				< 0.001
Married	4812 (63.39%)	4363 (62.78%)	449 (69.42%)	
Never married	1593 (19.21%)	1515 (20.08%)	78 (10.66%)	
Other	1646 (17.40%)	1472 (17.14%)	174 (19.91%)	
Poverty ratio, n (%)				0.465
< 1.3	2361 (19.67%)	2167 (19.87%)	194 (17.72%)	
≥ 1.3, < 3.5	2978 (34.57%)	2713 (34.51%)	265 (35.11%)	
≥ 3.5	2712 (45.76%)	2470 (45.62%)	242 (47.16%)	
Alcohol intake, n (%)				0.890
Yes	1666 (22.43%)	1523 (22.39%)	143 (22.75%)	
No	6385 (77.57%)	5827 (77.61%)	558 (77.25%)	
Smoking, n (%)				0.071
Current	2069 (26.98%)	1860 (26.60%)	209 (30.77%)	
Former	1789 (20.00%)	1632 (19.82%)	157 (21.74%)	
Never	4193 (53.02%)	3858 (53.58%)	335 (47.49%)	
Hypertension, n (%)				< 0.001
Yes	2551 (28.63%)	2254 (27.41%)	297 (40.60%)	
No	5500 (71.37%)	5096 (72.59%)	404 (59.40%)	
Vigorous activity, (n/%)				0.005
Yes	2098 (29.37%)	1949 (30.03%)	149 (22.87%)	
No	5953 (70.63%)	5401 (69.97%)	552 (77.13%)	
**Metabolic indicators**				
FBG (mg/dL)	100.16 ± 11.22	99.96 ± 11.12	102.08 ± 12.02	< 0.001
TG (mg/dL)	123.02 ± 99.67	122.20 ± 95.51	131.13 ± 133.60	0.013
HDL-C (mmol/L)	1.42 ± 0.43	1.43 ± 0.43	1.36 ± 0.40	0.002
HbA1c (%)	5.41 ± 0.38	5.40 ± 0.37	5.48 ± 0.39	< 0.001
Cr (mg/dL)	0.87 ± 0.24	0.87 ± 0.24	0.88 ± 0.20	0.137
eGFR	98.00 ± 19.45	98.35 ± 19.49	94.56 ± 18.64	< 0.001
UA (umol/L)	325.51 ± 80.50	324.79 ± 80.50	332.55 ± 80.12	0.065
ALB (g/L)	42.45 ± 3.25	42.51 ± 3.27	41.85 ± 3.09	0.001
RDW (%)	13.30 ± 1.23	13.29 ± 1.22	13.45 ± 1.30	0.001
**Insulin resistance indicator**				
TyG-WHtR	4.95 ± 0.93	4.92 ± 0.92	5.20 ± 0.91	< 0.001
TyG-BMI	244.40 ± 59.04	243.22 ± 59.02	256.03 ± 58.08	< 0.001
TyG-WC	838.43 ± 159.08	834.40 ± 158.95	877.85 ± 155.02	< 0.001
METS-IR	42.06 ± 11.21	41.83 ± 11.23	44.29 ± 10.75	< 0.001
HbA1c/HDL-C	4.15 ± 1.29	4.12 ± 1.29	4.37 ± 1.28	< 0.001
eGDR	8.33 ± 2.36	8.41 ± 2.34	7.53 ± 2.41	< 0.001

**Note:** BMI, body mass index; FBG, fasting blood glucose; TG, triglyceride; HDL-C, high density lipoprotein cholesterol; HbA1c, glycosylated hemoglobin A1c; Cr, creatinine; eGFR, estimate glomerular filtration rate; UA, uric acid; ALB, albumin; RDW, red blood cell distribution width; TyG-WHtR, triglyceride glucose-waist height ratio index; TyG-BMI, triglyceride glucose – body mass index; TyG-WC, triglyceride glucose – waist circumference; METS-IR, Metabolic score for Insulin resistance; HbA1c/HDL-C, the ratio of glycosylated hemoglobin A1c to high density lipoprotein cholesterol; eGDR, estimated Glucose Disposal Rate.

### Relationship between eGDR and kidney stones

**Fig 2:**
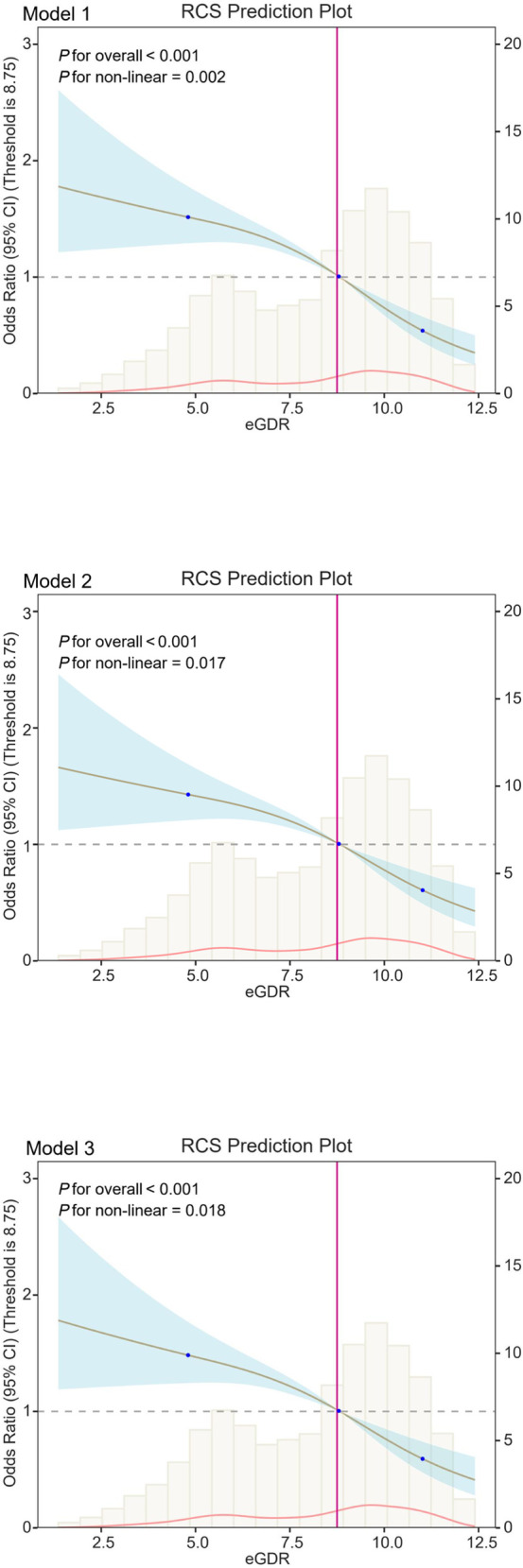
RCS shows a non-linear relationship between eGDR and kidney stones. The fitted regression line is a solid black line; the black dashed line indicates the position where the OR is equal to 1; the shaded area indicates the 95% CI; eGDR, estimated glucose disposal rate.

Table 2 shows the relationship between eGDR and the prevalence of kidney stones. In Model 1 (without any covariate adjustments), we observed an inverse association between eGDR and kidney stone risk (p < 0.05). A similar pattern emerged in Model 2, which adjusted for sociodemographic characteristics. Model 3 included the same adjustments as Model 2, with additional control for factors such as hypertension, alcohol intake, smoking, vigorous activity, eGFR, uric acid, TG, FBG, and dietary factors (calcium, sodium, and water). The adjusted OR for a per-unit increase in eGDR relative to kidney stone was 0.87 (95% CI: 0.61–1.26, *p* < 0.0001). We also stratified eGDR values into quartiles and examined their association with kidney stone incidence. The risk of developing kidney stones was significantly lower in the Q3 and Q4 groups compared to the Q1 group (*p* < 0.05). The odds ratio for kidney stone decreased with increasing eGDR (*p* for trend < 0.05). Furthermore, as shown in Fig 2, we found a negative correlation between eGDR and kidney stone risk (*p* for overall < 0.0001). These results suggest a nonlinear relationship between eGDR and kidney stone risk (*p* for nonlinear < 0.05).

**Table 2:** Association of eGDR with kidney stones, weighted.

	Model 1		Model 2		Model 3	
	OR (95%CI)	*p* value	OR (95%CI)	*p* value	OR (95%CI)	*p* value
eGDR	0.86 (0.83, 0.90)	< 0.0001	0.89 (0.85, 0.92)	< 0.0001	0.84 (0.78, 0.91)	0.0001
Categories						
Q1	Reference		Reference		Reference	
Q2	0.82 (0.63, 1.05)	0.118	0.88 (0.68, 1.15)	0.350	0.87 (0.61, 1.26)	0.463
Q3	0.51 (0.39, 0.66)	< 0.0001	0.56 (0.43, 0.73)	0.0001	0.54 (0.34, 0.85)	0.009
Q4	0.38 (0.29, 0.50)	< 0.0001	0.48 (0.36, 0.64)	< 0.0001	0.46 (0.28, 0.77)	0.004
*p* for trend	< 0.0001		< 0.0001		0.0008	

Model 1: Adjusted for no covariates. Model 2: Adjusted for gender, age, race, education, poverty ratio, and marital status. Model 3: Adjust for the variables in Model 2 plus hypertension, alcohol intake, smoking, vigorous activity, eGFR, uric acid, TG, FBG, Dietary (calcium, sodium, and water).

### Discriminative ability of different insulin resistance indices

In this study, we developed a ROC curve to assess the ability of six IR indicators to discriminate individuals diagnosed with kidney stones. The ROC curve analysis showed that eGDR demonstrated significantly better discriminatory power than TyG-BMI, TyG-WC, METS-IR, and the HbA1c/HDL-C ratio (Table 3). The area under the curve (AUC) values for TyG-WHtR, TyG-BMI, TyG-WC, METS-IR, HbA1c/HDL-C ratio, and eGDR were 0.591, 0.574, 0.586, 0.577, 0.554, and 0.604, respectively.

**Table 3:** ROC test of six alternative insulin resistance indicators for kidney stones.

Variables	AUC	95% CI	Cutoff value	Specificity	Sensitivity	*P* value	*P* value#
TyG-WHtR	0.591	(0.570, 0.612)	4.881	0.502	0.633	< 0.001	0.139
TyG-BMI	0.574	(0.552, 0.595)	236.653	0.517	0.599	< 0.001	0.001
TyG-WC	0.586	(0.565, 0.607)	804.120	0.459	0.676	< 0.001	0.036
METS-IR	0.577	(0.556, 0.599)	40.255	0.505	0.622	< 0.001	0.005
HbA1c/HDL-C	0.554	(0.532, 0.576)	4.118	0.537	0.555	< 0.001	< 0.001
eGDR	0.604	(0.583, 0.625)	9.083	0.473	0.696	< 0.001	–

*P* value#: comparation of eGDR with other five indicators.

### Subgroup analyses

**Fig 3:**
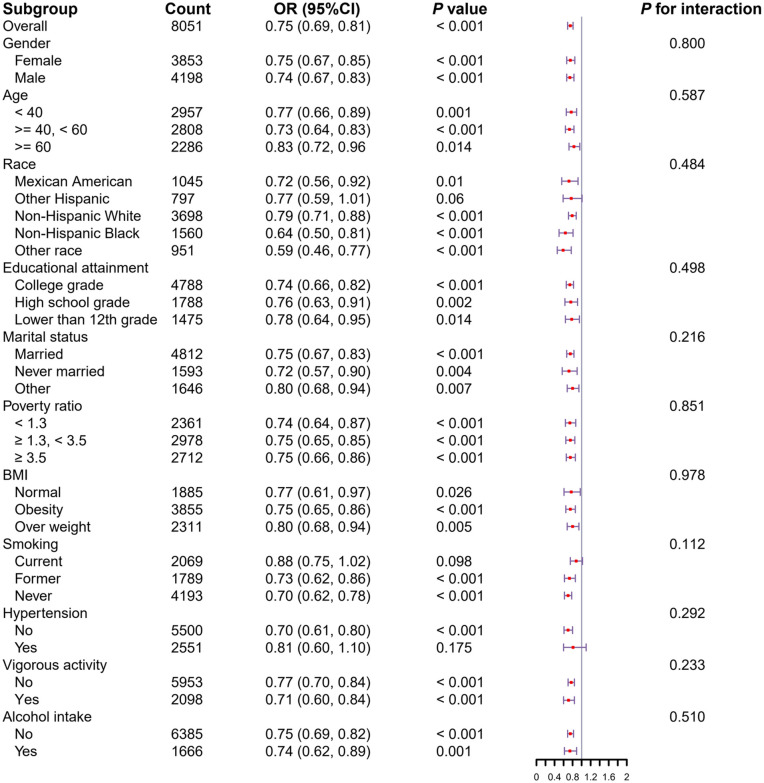
Subgroup analysis of the association between eGDR and kidney stones. The covariates including eGFR, uric acid, TG, FBG, as well as dietary factors such as calcium, sodium, and water.

We performed subgroup analyses to examine the relationship between eGDR and kidney stone risk, stratifying by sex, age, BMI, race, education, PIR, marital status, hypertension, alcohol consumption, smoking, and vigorous physical activity. No significant interactions were found in any subgroup, and eGDR consistently showed a negative correlation with kidney stone risk (Fig 3). These analyses incorporated covariates such as eGFR, uric acid, TG, FBG, and dietary factors (calcium, sodium, and water).

### Mediation effects of ALB and RDW on the association between eGDR and kidney stones

**Fig 4:**
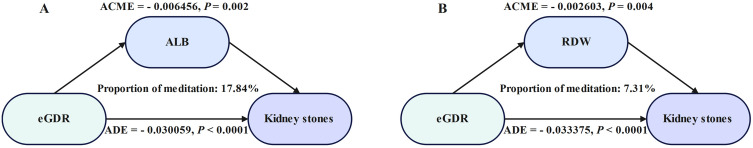
Mediation effects of ALB, RDW on eGDR to kidney stones association. Models were adjusted for gender, age, race, education, poverty ratio, marital status, hypertension, alcohol intake, smoking, vigorous activity, eGFR, uric acid, TG, FBG, Dietary (calcium, sodium, and water). Note: ACME, average causal mediation effects; ADE, average direct effects.

We conducted mediation analyses to explore the mediating roles of ALB and RDW in the relationship between IR and kidney stones. The mediating effects of ALB and RDW in the association between eGDR and kidney stone occurrence are illustrated in Fig 4. The mediation effect proportions for ALB and RDW were 17.84% and 7.31%, respectively (both *p* < 0.05). The mediation model adjusted for covariates, including gender, age, race, education, poverty ratio, marital status, hypertension, alcohol intake, smoking, vigorous activity, eGFR, uric acid, TG, FBG, and dietary factors (calcium, sodium, and water).

## Discussion

Our study demonstrates that eGDR is significantly associated with kidney stones. We found that eGDR negatively correlates with the risk of developing kidney stones, and this relationship persisted after adjusting for multiple covariates. Results from the RCS analysis further revealed a nonlinear association between eGDR and kidney stone risk. When comparing eGDR to other surrogate markers of insulin resistance – including TyG-WHtR, TyG-BMI, TyG-WC, METS-IR, and the HbA1c/HDL-C ratio – we observed a stronger association between eGDR and the risk of developing kidney stones. Additionally, as expected, our findings suggest that AIB and RDW partially mediate the relationship between eGDR and kidney stones.

Insulin resistance, a prevalent metabolic disorder, continues to draw considerable attention from both clinical and public health perspectives [26]. IR arises when key target tissues—such as liver, skeletal muscle, and adipose tissue – exhibit a diminished response to insulin signaling [27], impairing insulin’s ability to regulate blood glucose levels effectively. This dysfunction not only contributes to the progression of prediabetes and type 2 diabetes [28], but also plays a central role in the development of other metabolic disorders, including obesity, dyslipidemia, and non-alcoholic fatty liver disease (NAFLD) [29–31]. Although the hyperinsulinemic-euglycemic clamp remains the gold standard for quantifying insulin resistance, its technical complexity and high cost limit its widespread clinical use [32]. Therefore, alternative surrogate markers such as TyG-WHtR, TyG-WC, TyG-BMI, METS-IR, the HbA1c/HDL-C ratio, and eGDR have become essential tools for assessing insulin resistance in epidemiological and clinical research. Previous studies have linked poor glycemic control and IR to an increased risk of developing kidney stones. To explore this association further, we incorporated six surrogate markers of IR into our analysis to evaluate their relationships with kidney stone incidence in a non-diabetic population. Our findings are consistent with prior studies, demonstrating that elevated levels of TyG-BMI and METS-IR correlate with a higher risk of kidney stone [14,33]. In addition, our study contributes novel insights by identifying a negative association between eGDR - a relatively new IR indicator – and kidney stone risk. The ROC curve analysis revealed that eGDR had a stronger association between eGDR and the risk of developing kidney stones. Based on this superior performance, we selected eGDR for subgroup and mediation analyses. Subgroup analyses showed that no significant interactions were found in any subgroups, and additionally, eGDR consistently exhibited a negative correlation with kidney stone risks. These results further support the hypothesis that IR contributes to kidney stone formation and suggest that susceptibility may vary across demographic subpopulations. This variability highlights the need for IR markers with consistent predictive value across diverse groups. Our data suggest that the eGDR may serve as an indicator of IR, demonstrating a significant association with the risk of kidney stones in non-diabetic populations.

The mechanisms linking IR to kidney stone formation are multifaceted. First, IR may impair ammonia production by the kidneys, resulting in acidic urine. This acidic environment promotes the precipitation and aggregation of stone-forming components, such as uric acid and calcium oxalate, thereby increasing the risk of kidney stone development [34]. Second, IR has been shown to enhance the reabsorption of uric acid, raising serum uric acid levels and leading to hyperuricemia. Hyperuricemia is a well-established risk factor for kidney stone formation, particularly uric acid stones [35]. Third, IR may reduce urinary citrate levels, which in turn raises urinary calcium and uric acid concentrations, further promoting the formation of uric acid and calcium oxalate stones [36]. While these mechanisms provide insight into how IR may contribute to kidney stone formation, the exact pathophysiological processes remain unclear, and alterations in urinary composition due to IR are likely influential factors. To investigate potential alternative pathways, such as insulin resistance and oxidative stress pathways, we employed a mediated effects analysis model. Previous studies have demonstrated that IR can induce NAFLD [37], which impairs the liver’s ability to synthesize albumin. Reduced liver function leads to a decline in albumin production, subsequently lowering plasma albumin levels [38]. Additionally, IR is often associated with poor nutritional status, including obesity, which further impacts albumin synthesis [39]. Albumin plays an essential role in maintaining plasma colloid osmolality, facilitating transport functions, and acting as an antioxidant and acid-base buffer. Therefore, a decrease in albumin levels reduces the body’s ability to resist oxidative stress, potentially exacerbating the risk of kidney stone formation. Recent studies have shown that RDW correlates with systemic inflammation and oxidative stress, which tend to rise with increasing oxidative stress levels. Joosse HJ et al. [40] demonstrated that oxidative stress, independent of anemia or inflammation, alters erythrocyte morphology, leading to an elevated RDW. Several mechanisms have been proposed to explain this relationship: (1) Reactive oxygen species (ROS) can damage erythrocyte membranes through lipid peroxidation, as indicated by elevated malondialdehyde (MDA) levels, compromising membrane integrity and flexibility, which increases hemolysis and RDW [41]; (2) Oxidative stress interferes with erythropoiesis by activating nuclear factor kappa-B (NF-κB) and inhibiting nuclear factor erythroid 2-related factor 2 (Nrf2), disrupting erythroid progenitor differentiation [42]. The kidney, due to its high content of polyunsaturated fatty acids and oxygen sensors, is particularly vulnerable to oxidative and antioxidative imbalances. As they drive oxidative stress, ROS play a key role in the development of kidney stones [43]. Research has shown that reduced serum antioxidant levels lower the kidney’s antioxidant capacity, promoting oxidative stress and damage to the renal tubular epithelium, which can ultimately lead to kidney stone formation [44]. Based on this evidence, we hypothesize that oxidative stress mediates the relationship between IR and kidney stones. To test this hypothesis, we used a mediated effects analysis model to explore whether oxidative stress contributes to the link between IR and kidney stones. In our model, oxidative stress is indirectly represented by ALB and RDW levels. Our findings suggest that IR, potentially mediated by oxidative stress, increases the risk of kidney stone development.

### Study strengths and limitations

**Strengths.** (1) We used data from NHANES and applied appropriate NHANES sample weights, ensuring that our analysis accurately reflects the nationally representative U.S. population. (2) To enhance the reliability and generalizability of our results, we rigorously adjusted for multiple confounding covariates. (3) Notably, this study is the first to investigate the mediating roles of ALB and RDW in the association between eGDR and kidney stones within a nationally representative cohort, offering novel insights into potential underlying mechanisms.

**Limitations**. (1) The diagnosis of kidney stones relied on self-reported data from personal interviews, which may have introduced recall bias. (2) The NHANES database lacks detailed information on the specific types of kidney stones, limiting our ability to assess whether IR differentially affects various stone subtypes. (3) The cross-sectional design of our study precludes definitive conclusions about causality between IR, oxidative stress, and kidney stones. However, our findings support a possible link between IR and kidney stone formation, potentially mediated by oxidative stress, and lay the groundwork for future causal investigations. (4) Despite adjusting for numerous confounding factors, residual confounding from unmeasured variables cannot be completely ruled out. Therefore, it is critical that future research validate our findings through longitudinal cohort studies and mechanistic ex vivo experiments.

## Conclusion

This study reveals that a negative correlation exists between eGDR and kidney stones. Additionally, the relationship between IR and kidney stones is mediated by ALB and RDW. These findings suggest that reducing chronic inflammation and oxidative stress may help prevent kidney stone formation, providing evidence-based health recommendations for kidney stone prevention.

## Supporting information

S1 Data Datas. (XLSX)
